# Frailty phenotype, genetic risk and long-term incident systemic lupus erythematosus risk: Insights from the UK Biobank study integrated with multi-omics analysis

**DOI:** 10.1016/j.clinme.2026.100598

**Published:** 2026-06-04

**Authors:** Peizhi Deng, Siyu Yan, Jianyun Lu

**Affiliations:** a Department of Dermatology, The Third Xiangya Hospital, Central South University, Tongzipo Road, Changsha, Hunan 410008, China; b Medical Ozone Research Center, The Third Xiangya Hospital, Central South University, Tongzipo Road, Changsha, Hunan 410013, China

**Keywords:** Frail, SLE, PRS, Peripheral inflammation markers, Proteomics

## Abstract

**Background:**

Frailty, a marker of biological ageing and reduced physiological reserve, is linked to many chronic conditions, yet its role in the development of systemic lupus erythematosus (SLE) remains unclear.

**Aims:**

To examine the association between frailty and incident SLE and to investigate potential inflammatory and genetic mechanisms underlying this relationship.

**Methods:**

We conducted a prospective cohort study among 462,762 participants from the UK Biobank to examine the associations of physical frailty and frailty index with incident SLE. Cox proportional hazards models were used to estimate hazard ratios and 95% confidence intervals, with further analyses assessing the interaction and joint effects of frailty and polygenic risk. Gene-level overlap between frailty and SLE was evaluated using MAGMA. In addition, we integrated circulating inflammatory markers and plasma proteomic profiles to identify potential mediating pathways and candidate biomarkers.

**Results:**

During a median follow-up of 13.2 years, 309 of 462,762 participants (0.07%) developed incident SLE. Compared to non-frail individuals, both pre-frail and frail participants had a higher risk of developing SLE, with stronger associations observed in those with higher polygenic risk scores. Gene analysis identified 39 shared genes that were significantly correlated with both frailty and SLE. Circulating inflammatory markers, including C-reactive protein, were found to partially mediate the frailty-SLE relationship. Proteomic analyses identified ANGPTL3, CST3 and TNFSF8 as the candidate risk-related biomarkers associated with future incident SLE.

**Conclusion:**

Frailty is associated with an increased long-term risk of incident SLE, particularly in genetically susceptible individuals. Inflammatory cell measures and circulating proteins, including ANGPTL3, CST3 and TNFSF8, may help identify elevated SLE risk in pre-frail and frail populations.

## Highlights

• Both physical frailty and frailty index can increase long-term risk of incident SLE.
• Higher risk was observed in individuals with greater genetic predisposition compared to their counterparts.
• CRP level was the principal inflammatory marker that mediated this association.
• ANGPTL3, CST3 and TNFSF8 are the candidate risk-related biomarkers associated with incident SLE in frail individuals.

## Graphical abstract

**Figure:**
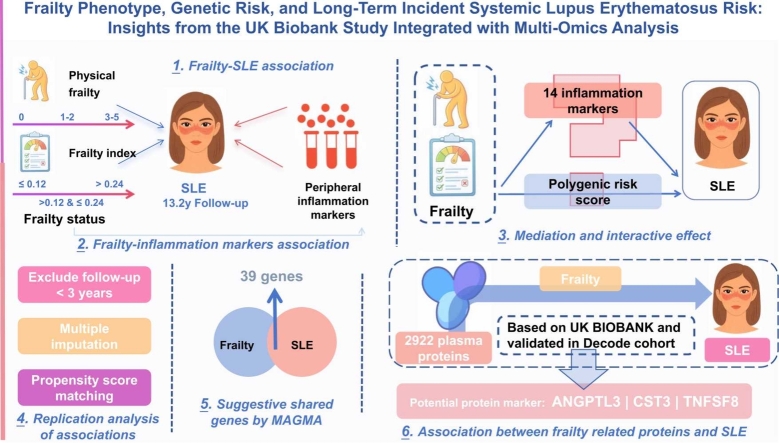

## Introduction

Systemic lupus erythematosus (SLE) is a chronic, systemic autoimmune disease characterised by immune-mediated inflammation and multi-organ involvement. Its clinical course is typically protracted and fluctuating, and its pathogenesis reflects a complex interplay of innate and adaptive immune dysregulation, aberrant inflammatory signalling pathways and genetic susceptibility.^1^ SLE can substantially impair quality of life and organ function and is associated with adverse long-term outcomes, affecting millions of individuals worldwide.^2^

Frailty, a clinical syndrome reflecting biological ageing and reduced physiological reserve, is prevalent in middle-aged and older adults and is consistently associated with a broad spectrum of adverse outcomes, including mortality, hospitalisation and functional decline. Frailty is often accompanied by chronic, low-grade systemic inflammation (ʻinflammaging’) and immune dysregulation, manifested by elevated pro-inflammatory cytokines and diminished stress-response capacity, suggesting that inflammatory processes play a central role in the development and progression of frailty.^3^

In recent years, frailty has attracted growing interest in the context of immune-mediated diseases, with existing evidence largely focusing on their prognostic implications among individuals with established SLE. Multiple studies have shown that, in patients with SLE, frailty is associated with greater accumulated organ damage, poorer health-related quality of life, and higher risks of hospitalisation and readmission, indicating that frailty may serve as an independent predictor of adverse outcomes in SLE.^3^, ^4^, ^5^ However, the biological mechanisms linking frailty to SLE remain insufficiently defined, particularly with respect to whether and how inflammatory pathways mediate this relationship and whether such mediation is causal.^6^ Beyond inflammation, SLE has a well-established genetic basis and notable familial aggregation.^7^ Given that SLE is influenced by numerous risk loci, each with modest effects, polygenic risk scores (PRS) have been developed to quantify the cumulative contribution of multiple variants.^8^, ^9^, ^10^ Recent studies have advanced understanding of gene–environment interactions relevant to SLE risk, which may help explain disease pathogenesis and clinical heterogeneity.^8^, ^9^ To date, however, no study has systematically evaluated the interaction between genetic susceptibility and frailty in relation to SLE risk.

In this study, we aimed to investigate the prospective associations of physical frailty and the frailty index with incident SLE among 462,762 UK Biobank participants. We further examined whether genetic susceptibility modified these associations and evaluated the joint effects of frailty and polygenic risk on SLE risk. In addition, we integrated circulating inflammatory markers and multi-omics plasma proteomic data to identify candidate pathways and biomarkers that may mediate the frailty–SLE relationship.

## Materials and methods

### UK Biobank cohort

The UK Biobank is a large-scale, population-based prospective study, with detailed descriptions of its design and data collection available in previous publications.^11^ In brief, the cohort includes over 500,000 adults aged between 37 and 73 years, who were recruited from 22 assessment centres across the UK between 2006 and 2010. At the time of enrolment, participants completed a touchscreen survey, underwent standardised physical assessments and provided biological samples. For this analysis, individuals who withdrew consent, had SLE at the baseline or had missing or uncertain responses to essential variables were excluded. A summary of the inclusion and exclusion criteria can be found in Fig. S1. The study was approved by the North West Multi-centre Research Ethics Committee (11/NW/0382), and all participants provided written informed consent. Data access was granted under application number 224336.

### Assessment of physical frailty and frailty index

Baseline frailty was evaluated using two complementary methods: physical frailty and a frailty index.^12^ For physical frailty, we applied Fried’s phenotype model using variables from the UK Biobank, following established protocols.^13^ Five components were included: unintentional weight loss, self-reported exhaustion, low physical activity, slow walking speed and reduced grip strength. Participants were given a score ranging from 0 to 5. Based on this score, individuals were categorised as non-frail (no components), pre-frail (one or two components) or frail (at least three components).^13^ The frailty index was derived from 49 self-reported health deficits, encompassing chronic conditions, functional limitations and mental health, in line with a previously published UK Biobank specification.^14^ Each deficit was marked as either present (1) or absent (0). For participants with fewer than 10 missing items, the index was calculated as the proportion of present deficits among those assessed (ranging from 0 to 1), with higher values reflecting greater frailty. Participants were classified as non-frail (index ≤0.12), pre-frail (0.12 <index ≤0.24), or frail (index >0.24).^14^ Frailty was assessed at baseline recruitment only and was treated as a fixed exposure in the subsequent analyses. Therefore, the Cox models estimated the association between baseline frailty status and incident SLE, rather than the effect of longitudinal changes in frailty over time.

### Ascertainment of SLE

The diagnosis of SLE was primarily confirmed through clinical data obtained from hospital admission records and primary care, with additional information gathered from self-reported physician diagnoses. Additional cases were identified through supplementary sources. ICD-10 code M32 was used to establish the diagnosis. All diagnoses were verified via nurse-administered verbal interviews and cross-referenced with primary care and inpatient records.^15^

### Covariates

Sociodemographic factors included age, sex, ethnicity (White or other), Townsend deprivation index, employment status (employed, retired or other), and educational level (below or above college degree). Participants who selected ʻNone of the above’ or ʻPrefer not to answer’ were excluded. Lifestyle factors encompassed smoking status and alcohol consumption (never, former or current). Trained nurses measured participants’ height and weight, from which body mass index (BMI) was calculated. The PRS for SLE was obtained from the precomputed UK Biobank Standard PRS resource (Data-Field 26278), rather than being newly derived in the present study.^10^ This field provides a continuous relative-risk PRS for SLE generated within the UK Biobank PRS Release framework using external GWAS training data only. Triglyceride level was measured using blood samples collected during UK Biobank assessment centre visits. Further details regarding the protocols can be found on the UK Biobank website (https://www.ukbiobank.ac.uk). The annual average concentrations of particulate matter with aerodynamic diameter ≤2.5 μm (PM_2.5_) were estimated using a Land Use Regression model developed by the European Study of Cohorts for Air Pollution Effects project. Model 1 included sex, age, ethnicity and Townsend deprivation index, while Model 2 accounted for Model 1 plus BMI, employment status, education level, smoking status, alcohol consumption, triglyceride levels and PM_2.5_ exposure.

### Peripheral inflammation markers

At baseline, haematological data collected during recruitment were used to derive 14 systemic inflammation markers: C-reactive protein (CRP), platelet count, total leucocyte count, and both absolute counts and proportions of basophils, eosinophils, lymphocytes, monocytes and neutrophils. In addition, the neutrophil-to-lymphocyte ratio was calculated as an indicator of overall inflammatory status. To normalise the distribution of the neutrophil-to-lymphocyte ratio, it was log-transformed. Detailed assay protocols can be found on the UK Biobank website.

### Plasma proteomic profiling

Plasma proteomic data were obtained from the UK Biobank Pharma Proteomics Project (UKB-PPP), which assayed samples from 54,219 participants using the Olink Explore 3072 platform. Although the UKB-PPP resource includes a small subset of samples collected at later visits, the present proteomic analysis was restricted to plasma samples obtained at the baseline assessment-centre visit at recruitment. The assay targets 2,923 proteins across eight prespecified panels spanning cardiometabolic, inflammatory, neurological and oncologic domains (two panels per domain). Because GLIPR1 had >80% missingness, it was excluded, leaving 2,922 proteins for analysis. Whole blood was collected in 9 mL EDTA tubes and processed into 850 µL aliquots to separate plasma, buffy coat and red blood cells. Plasma was stored at −80 °C and shipped on dry ice to Olink (Sweden) for profiling. Protein abundances were reported as Normalized Protein eXpression (NPX) values. Further details on participant selection, quality control procedures and NPX generation have been described previously.^16^ The full protein list and panel assignments are provided in Table S1.

### Statistical analysis

#### Associations between physical frailty and SLE risk

Descriptive statistics were summarised for participants with and without incident SLE. Continuous variables are presented as mean (SD), and categorical variables as number (%). Group differences were evaluated using Kruskal–Wallis tests for continuous measures and χ² tests for categorical variables. Cumulative incidence by frailty status was visualised using Kaplan–Meier curves. Cox proportional hazards models were used to estimate hazard ratios (HRs) and 95% confidence intervals (CIs) for the association between frailty and incident SLE, with follow-up time as the underlying time scale. Frailty was analysed both as a categorical exposure (non-frail, pre-frail, frail) and as a continuous measure (per 1-point increase in the physical frailty phenotype score and per quartile increase in the frailty index). The proportional hazards assumption was assessed using Schoenfeld residuals and was not violated. Potential dose–response relationships were examined using restricted cubic splines with four knots placed at the 5th, 35th, 65th and 95th percentiles.^17^ In addition, we evaluated the joint association of frailty and PRS with incident SLE by cross-classifying participants into six groups according to frailty level (low/high) and genetic risk category (low/intermediate/high). HRs for all five non-reference groups were estimated within the same Cox proportional hazards model, using the low genetic risk/low frailty group as the reference category. Thus, the analysis included not only the concordant high-risk/high-frailty group but also the discordant groups, such as low genetic risk/high frailty and high genetic risk/low frailty. Multiplicative interaction was assessed by including the cross-product term between frailty and genetic risk in the model.

#### Replication analysis of associations

We conducted several sensitivity analyses to assess the robustness of the primary findings. First, to reduce the possibility of reverse causation and capture longer-term associations, we repeated the analyses after excluding participants who developed SLE within the first 3 years of follow-up. Second, to address potential bias arising from incomplete covariate data, we performed multiple imputations for missing baseline covariates under the missing at random assumption.^18^ Imputation was applied only to covariates with incomplete data and was not used for the primary exposure variables, incident SLE outcome or follow-up time. Third, to further limit confounding, we applied propensity score matching (PSM) to construct a matched cohort and re-estimate the main associations. PSM was implemented using nearest-neighbour greedy matching at a 1:4 ratio, without replacement, with a caliper of 0.4. Propensity scores were derived from sex, age, ethnicity, Townsend deprivation index, body mass index, employment status, educational attainment, smoking status, alcohol intake, triglycerides and PM_2.5_ level.

#### Gene analysis by MAGMA

Gene-level analyses were performed with MAGMA software, which used Phase 3 data from the 1000 Genomes Project to combine single nucleotide polymorphism (SNP)-level association data into gene scores. This method assessed the association between each gene and the phenotype.^19^ For more information on the methodology and settings, please consult the MAGMA documentation.^19^ Summary statistics for physical frailty were obtained from a recent UK Biobank genome-wide association study of 175,226 European-ancestry participants.^20^ SLE summary statistics were derived from the FinnGen consortium datasets, which excluded UK Biobank samples, comprising 835 cases and 299,327 controls (Table S2).

#### Mediating effects of inflammatory markers

We assessed the relationship of frailty with each of the 14 inflammatory markers using separate linear mixed-effects models. In each model, frailty and all covariates entered as fixed effects, and the inflammatory marker served as the outcome variable. From these models, we extracted standardised β coefficients and converted them to Cohen’s d following established methods.^21^ Within the same framework, we then evaluated the direct association between each inflammatory marker and SLE onset using Cox proportional-hazards models.

To test mediation, we applied the ʻmediation’ package in R (version 4.4.3) to implement a three-variable path analysis of frailty → inflammation → SLE. Frailty–inflammation links were modelled by linear regression, while frailty–SLE and inflammation–SLE paths used survival regression.^22^ Mediation effects were judged significant via 5,000 bootstrap replications. All models adjusted for the same covariates, and p-values were corrected for multiple comparisons using the Benjamini–Hochberg false-discovery-rate (FDR) approach.

#### Association between frailty-related proteins and SLE

To investigate the relationship between frailty-related proteins and SLE, we employed a three-step association framework. In the first step, logistic regression models, adjusted for relevant covariates, were used to identify proteins associated with physical frailty and the frailty index. In the second step, Cox proportional hazards models were applied to examine the association between frailty-related circulating proteins and SLE incidence. The third step involved proteome-wide Mendelian randomisation (MR) to validate the associations observed in step two. Proteins were considered potential modulators of SLE only if consistent associations were identified across all three steps.

Protein instruments for MR were obtained from the UKB-PPP and DeCODE genetics.^23^ Independent SNPs (cis-pQTLs) significantly associated with plasma protein levels (P < 5 × 10^−8^) and located within 1 Mb of the encoding gene were selected. Independence among SNPs was ensured using the 1000 Genomes European reference panel, applying linkage disequilibrium filtering (r² < 0.001, clumping window >5,000 kb). For proteins with only one valid instrument, we applied the Wald ratio method; for those with multiple instruments, the inverse variance–weighted MR (MR-IVW) method was used.^24^ Effect estimates were reported as odds ratio per SD increase in protein levels. Where a sufficient number of independent instruments was available, we additionally conducted MR sensitivity analyses using complementary estimators and tests for heterogeneity and horizontal pleiotropy.^25^ For proteins instrumented by only one cis-pQTL, conventional sensitivity analyses were either not estimable or not statistically informative; therefore, these MR results were interpreted cautiously as supportive genetic evidence rather than definitive proof of causality. All statistical tests were two-sided, adjusted for multiple comparisons using the Benjamini–Hochberg procedure, and significance was defined as FDR <0.05. The GeneMANIA platform was used to access diverse datasets, including genetic interactions, pathways and co-expression data for the target genes.^26^ By integrating multiple data sources and interaction networks, this platform provides valuable insights into the potential biological roles of these genes.^27^

## Results

### Frailty and risks of incident SLE

**Fig. 1:**
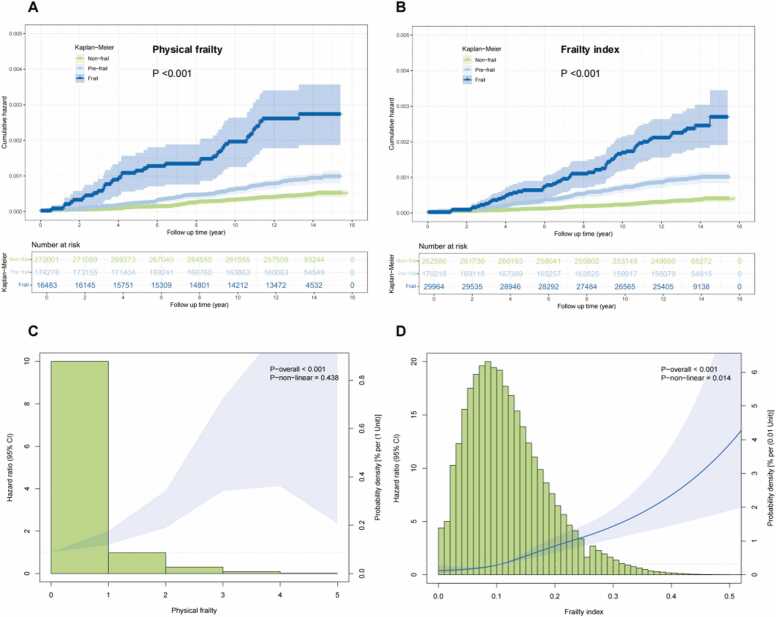
Cumulative (A, B) and dose–response (C, D) risks of incident SLE in accordance with frailty profile. Model were adjusted for age, sex, Townsend deprivation index, ethnicity, body mass index, education level, employment status, smoking status, alcohol drinking status, triglycerides, PM_25_ level. Levels of significance: P < 0.05 (log-rank test). Note: CI, confidence interval; HR, hazard ratio.

**Fig. 2:**
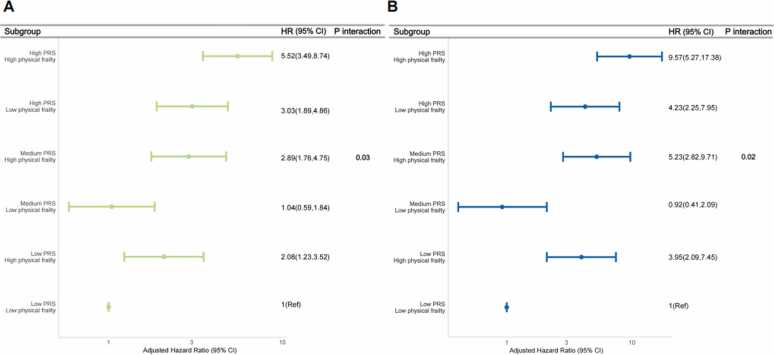
Joint effects of (A) Physical frailty, (B) Frailty index and PRS on the risk of incident SLE, respectively. Frailty was classified into high and low levels based on the median, and genetic risk was classified into low, intermediate and high categories, yielding six joint groups. Participants with low genetic risk and low frailty were used as the referent category. Hazard ratios for the other five groups were estimated within the same Cox model, including discordant groups such as low genetic risk/high frailty and high genetic risk/low frailty. Models were adjusted for Model 1. Multiplicative interaction was evaluated by including the cross-product term between frailty and genetic risk in the model, and P-interaction <0.05 was considered statistically significant.

Over a median follow-up of 13.2 years (IQR 12.8–14.2), 309 participants (0.07%) among 462,762 participants developed SLE (Table 1 and Fig. S1). The analytical cohort was predominantly White; according to baseline characteristics, 91.3% of both non-SLE and incident SLE participants were White. Based on incident cases and overall participants, the crude incidence was approximately 5.1 per 100,000 person-years. Kaplan–Meier curves revealed significantly different cumulative SLE incidence across the non-frail, pre-frail and frail groups (log-rank p < 0.001; Fig. 1A and B). In both partially and fully adjusted Cox models (Table 2), pre-frail individuals had an elevated risk of SLE compared with non-frail individuals, with HRs of 1.80 (95% CI 1.33,2.44) for physical frailty and 2.78 (95% CI 1.98,3.91) for the frailty index. Frail participants had HRs of 5.92 (95% CI 4.26,8.22) and 8.49 (95% CI 5.64,12.80), respectively. When frailty was modelled continuously, each one-unit increase in physical frailty and the frailty index corresponded to HRs of 1.60 (95% CI 1.39,1.85) and 1.86 (95% CI 1.60,1.97), respectively (Table 2). These associations remained robust in extensive sensitivity analyses (Tables S3–S5). Only the dose–response curve of frailty index demonstrated a non-linear relationship (*P* for non-linearity <0.001, Fig. 1C and D). Fig. 2 and Table S6 present the joint effects of genetic susceptibility and frailty on SLE risk across six combined groups defined by frailty level and PRS category. Compared with the low genetic risk/low frailty reference group, hazard ratios were estimated separately for the remaining five groups, including discordant categories such as low genetic risk/high frailty and high genetic risk/low frailty. The highest risk of SLE onset was observed among participants with both high genetic risk and high frailty. A statistically significant multiplicative interaction between frailty and genetic risk was also observed (P for interaction <0.05).

**Table 1:** Baseline characteristics of participants categorised by SLE and Non-SLE among UK biobank populations.

Characteristic	Level	Non-SLE (N = 462,453)	SLE (N = 309)	*P*-value
Age, years		56.5 (8.1)	57.8 (7.7)	0.004
Sex	Female	251,174 (54.3)	242 (78.3)	<0.001
	Male	211,279 (45.7)	67 (21.7)	
Ethnicity	Others	40,207 (8.7)	27 (8.7)	0.793
	White	422,246 (91.3)	282 (91.3)	
Townsend deprivation index		−1.37 (3.05)	−0.83 (3.08)	0.002
Smoking status	Never	252,558 (54.8)	143 (46.6)	0.005
	Previous	160,790 (34.9)	119 (38.8)	
	Current	47,703 (10.3)	45 (14.7)	
Alcohol drinker status	Never	18,978 (4.1)	16 (5.2)	0.001
	Previous	16,010 (3.5)	22 (7.1)	
	Current	427,116 (92.4)	270 (87.7)	
Body mass index, kg/m^2^		27.39 (4.76)	27.81 (5.34)	0.129
Employment status	Retired/Others	190,255 (41.5)	168 (55.8)	<0.001
	Employed	268,454 (58.5)	133 (44.2)	
Education level	Others	40,207 (8.7)	27 (8.7)	0.998
	University/college or above	422,246 (91.3)	282 (91.3)	
Triglycerides, mmol/L		1.74 (1.03)	1.74 (1.15)	0.989
Polygenic risk score		0.10 (0.99)	0.66 (1.16)	<0.001
PM_25_, µg/m³		9.98 (1.05)	10.09 (1.00)	0.077
Physical frailty		0.58 (0.83)	1.07 (1.13)	<0.001
Frailty index		0.12 (0.07)	0.17 (0.08)	<0.001
By physical frailty	Non-frail	271,880 (58.8)	121 (39.2)	<0.001
	Pre-frail	174,130 (37.7)	148 (47.9)	
	Frail	16,443 (3.6)	40 (12.9)	
By frailty index	Non-frail	262,491 (56.8)	89 (28.8)	<0.001
	Pre-frail	170,063 (36.8)	155 (50.2)	
	Frail	29,899 (6.5)	65 (21.0)	

Note: Continuous variables were presented as mean (SD). Categorical variables were presented as number (percentage).

**Table 2:** Associations between frailty and risk of SLE.

Exposure		Model	HR (95% CI)	*P*-value
Physical frailty	Non-frail	1	Ref	Ref
	Pre-frail		2.08 (1.53,2.84)	<0.001
	Frail		4.05 (2.28,7.22)	<0.001
	Per 1 point increase		1.58 (1.43,1.75)	<0.001
	Non-frail	2	Ref	Ref
	Pre-frail		1.80 (1.33,2.44)	<0.001
	Frail		4.05 (2.37,6.93)	<0.001
	Per 1 point increase		1.60 (1.39,1.85)	<0.001
Frailty index	Non-frail	1	Ref	Ref
	Pre-frail		2.55 (2.00,3.32)	<0.001
	Frail		5.92 (4.26,8.22)	<0.001
	Per 1 quartile increase		1.76 (1.57,1.97)	<0.001
	Non-frail	2	Ref	Ref
	Pre-frail		2.78 (1.98,3.91)	<0.001
	Frail		8.49 (5.64,12.80)	<0.001
	Per 1 quartile increase		1.86 (1.60,1.97)	<0.001

Note: CI, confidence interval; HR, hazard ratio.

### Gene analysis of MAGMA

Gene-level analysis using MAGMA identified 437 genes significantly associated with frailty (FDR <0.05), as shown in Table S7. Additionally, 102 genes were found to be significantly associated with SLE (Table S8). Ultimately, 39 genes were shared between frailty and SLE based on the MAGMA analyses.

### The mediating effect of inflammatory markers

**Fig. 3:**
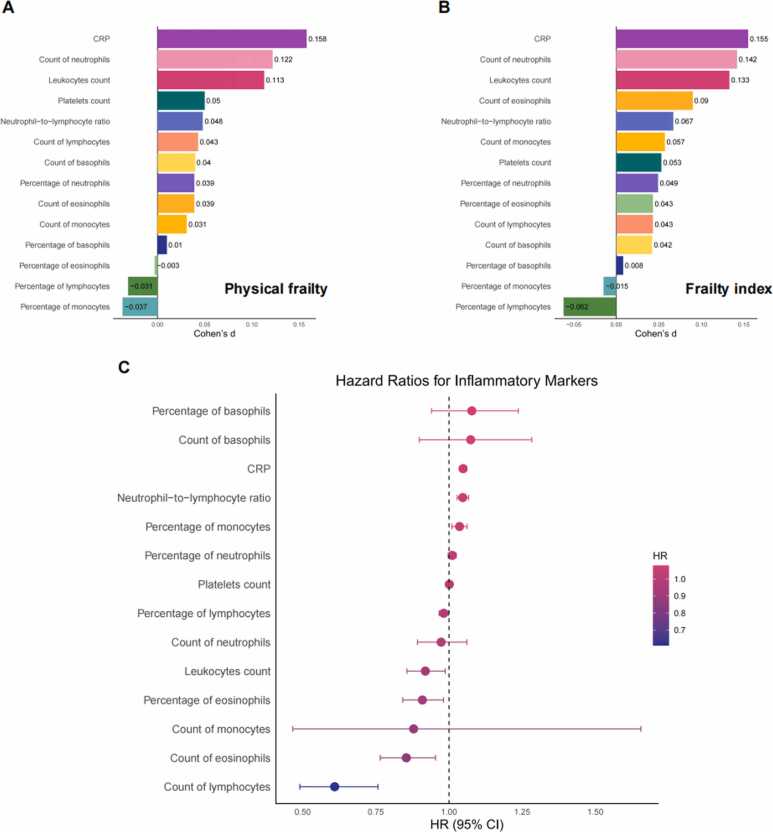
Associations between frailty severity, inflammation markers, and SLE. (A, B) All inflammatory markers showed significant associations with physical frailty and frailty index. All P values were two-sided and unadjusted. (C) Cox proportional hazards models provided evidence between 12 inflammatory markers and incident SLE.

13 and 14 inflammatory markers remained significantly associated with both physical frailty and the frailty index, respectively, after adjusting for covariates and correcting for multiple comparisons (Fig. 3A and B; Table S9). Serum CRP exhibited the largest effect size (Cohen’s d = 0.158 for physical frailty; Cohen’s d = 0.155 for the frailty index). Seven markers – including CRP, leucocyte count, eosinophils, count of lymphocytes, percentage of monocytes, and neutrophil-to-lymphocyte ratio – were independently associated with incident SLE (FDR <0.05) (Fig. 3C; Table S10).

Mediation analysis revealed that six markers partially mediated the association between physical frailty and SLE, while eight markers partially mediated the association between frailty index and SLE (Fig. S2; Table S11). The proportion of mediated variance for significant mediators ranged from 0.8% (neutrophil-to-lymphocyte ratio) to 14.5% (CRP levels) for physical frailty, and from 1.5% (percentage of monocytes) to 13.5% (CRP levels) for the frailty index.

### Frailty-related proteins and the risk of SLE

**Fig. 4:**
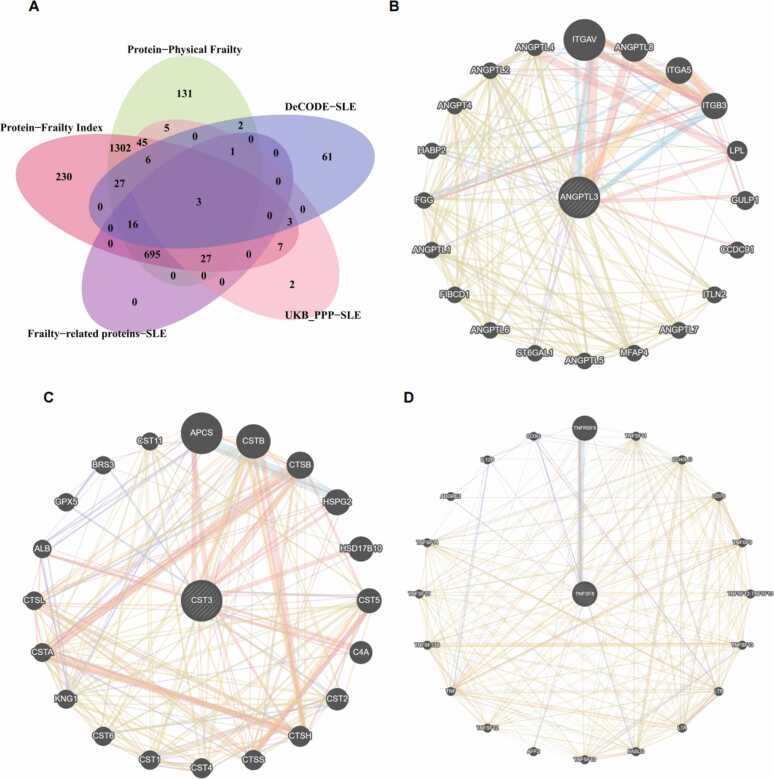
Relationship between plasma proteins, frailty, and SLE. (A) Venn diagram of frailty-related and SLE-related plasma proteins. (B) GeneMania gene network of ANGPTL3 as the core. (C) GeneMania gene network of CST3 as the core. (D) GeneMania gene network of TNFSF8 as the core.

We first identified proteins associated with physical frailty and frailty index separately, as shown in Tables S12–S13. A total of 2,121 proteins were significantly associated with both frailty measures. These 2,121 shared frailty-associated proteins were then carried forward into the subsequent protein–SLE analysis, in which 741 proteins showed significant associations with incident SLE (Table S14). Therefore, the 741 proteins were not derived from a different dataset, but represented a subset of the 2,121 proteins shared by the two frailty measures. Next, proteome-wide MR was used to validate these results and support a potential causal role between three proteins and SLE, with consistent effect directions observed in the IVW method (Tables S15–S16). For ANGPTL3, CST3 and TNFSF8, additional sensitivity analyses were performed to evaluate the robustness of the IVW estimates, yielding consistent results (Table S17). Cochran’s Q and MR-PRESSO tests detected no evidence of heterogeneity or outliers and no appreciable horizontal pleiotropy (P > 0.05). Taken together, these analyses identified ANGPTL3, CST3 and TNFSF8 as candidate risk-related biomarkers associated with future SLE development (Fig. 4A). The GeneMANIA analysis revealed potential gene networks that could be interactive, with ANGPTL3 positioned at the centre, as illustrated in Fig. 4B. Notably, the networks linked to ANGPTL3 predominantly show enrichment in pathways related to acylglycerol regulation, integrin complexes and lipid homoeostasis (Table S18). Another network, with CST3 at its core, is depicted in Fig. 4C. This network primarily emphasises functional pathways related to the regulation and activity of enzymes, sensory functions and structural components (Table S19). Furthermore, the gene interaction network centred around TNFSF8, shown in Fig. 4D, exhibits significant enrichment in pathways related to immune cell activation and differentiation, as well as apoptosis and inflammatory response (Table S20).

## Discussion

This study, integrating large-scale cohort data with genetic analyses, highlights an increased SLE risk among pre-frail and frail individuals, independent of potential confounders, with stronger effects observed in genetically predisposed populations. Multiple inflammatory markers, particularly CRP, partially mediated the association between frailty and SLE. Moreover, ANGPTL3, CST3, and TNFSF8 were identified as candidate risk-related biomarkers associated with future SLE development in frail individuals, suggesting inflammatory pathways as a key mechanism linking frailty to SLE.

Our findings build on existing work by leveraging data from 462,762 UK Biobank participants with extended follow-up, allowing robust time-to-event modelling and thorough control for confounding. This advances prior research that has been mainly cross-sectional or restricted to disease cohorts, by showing that frailty predicts new-onset SLE risk in a general population context. Of note, we observed a multiplicative interaction between frailty and PRS, such that individuals with both high frailty and elevated genetic risk had substantially greater SLE incidence than expected from their individual effects. This interaction highlights the epidemiological and clinical importance of considering both genetic predisposition and biological vulnerability in SLE risk stratification.

Consistent with mechanistic hypotheses, immune and inflammatory dysregulation likely underlie the frailty–SLE link. SLE pathogenesis is characterised by chronic systemic inflammation, autoantibody production and dysregulated immune signalling, with proteomic profiling identifying clusters of patients with distinct inflammatory and clinical features.^28^, ^29^ Frailty is similarly characterised by low‑grade systemic inflammation (ʻinflammaging’) and impaired immune competence, manifesting as elevations in inflammatory biomarkers and reduced physiological reserve.^30^ These shared inflammatory characteristics support a model in which systemic immune dysregulation contributes both to frailty and the initiation or propagation of autoimmune processes that culminate in SLE.

In line with this, our mediation analyses suggest that circulating inflammatory markers partially explain the association between frailty and SLE. This likely reflects both the broad range of factors influencing frailty and the multifactorial aetiology of SLE. Inflammation should therefore be viewed as an important but not exclusive pathway linking frailty to autoimmune disease emergence. Other biological systems – including metabolic dysregulation, cellular senescence and immune cell functional shifts – may also contribute. For example dyslipidaemia, which is prevalent in SLE and associated with increased inflammatory burden and organ involvement, may interface with frailty‑related immune dysfunction to elevate disease risk.^31^ Cellular senescence promotes chronic inflammation through the senescence-associated secretory phenotype, which has been implicated in autoimmune diseases like SLE.^32^ Additionally, immune cells in SLE exhibit altered metabolism and functional shifts that drive sustained immune activation and tissue damage, further linking metabolic dysfunction to autoimmunity.^33^

Type I interferon pathway activation is a well-established feature of SLE pathogenesis and warrants explicit consideration in interpreting our findings.^34^, ^35^ The fact that canonical interferon-related proteins did not emerge among our final candidates should not be interpreted as evidence against the importance of interferon biology in SLE. A more likely explanation is methodological: our analysis focused on future incident SLE in a population-based cohort, rather than on cross-sectional disease activity in clinically manifest SLE; in addition, interferon activation is often captured more sensitively by IFN-stimulated gene signatures than by single circulating proteins, and stringent multiple-testing correction may further reduce sensitivity to detect proteins with smaller effect sizes.^36^, ^37^, ^38^ Therefore, our Olink-based results should be interpreted as exploratory and complementary, rather than as a complete representation of established SLE immunopathology.

Among the identified proteins, ANGPTL3 is biologically plausible because, beyond its canonical role in lipid metabolism, it has documented links to endothelial biology and inflammatory signalling, including regulation of IL-1β/NF-κB-related pathways.^39^ CST3 (cystatin C) is the most directly supported by prior SLE literature: elevated cystatin C has been reported in SLE and has been associated with inflammation, proteinuria, anti-dsDNA positivity, lower C3 and higher disease activity, suggesting that it may reflect inflammatory and organ-damage pathways rather than renal filtration alone.^40^ TNFSF8 (CD30L) is less clearly established in SLE, but the CD30/CD30L axis is involved in T-cell and B-cell communication, and prior studies have reported increased CD30-related immune activation in SLE, supporting at least biological plausibility.^41^ Nonetheless, these proteins should be regarded as candidate signals requiring external and functional validation, particularly given the exploratory nature of large-panel proteomics.

Several limitations should be considered. First, frailty measures relying on self-reported components may introduce recall bias and misclassification, though such approaches are standard in large observational studies and essential for scalable frailty assessment. Meanwhile, frailty was assessed only at baseline recruitment. As a result, we were unable to evaluate longitudinal changes in frailty status or examine whether frailty trajectories over time were associated with incident SLE. Therefore, the reported hazard ratios reflect baseline frailty status rather than time-varying frailty patterns. Future studies incorporating repeated frailty assessments are needed to determine whether dynamic changes in frailty further influence SLE risk. Second, the analytical cohort was predominantly White, which limits the generalisability of our findings to more ethnically diverse populations. This is important because SLE disproportionately affects some minority ethnic groups and often presents at younger ages than those represented in our cohort. Therefore, the absolute incidence observed in this study may not fully reflect that of the general population. Further validation in more ethnically diverse cohorts is warranted. Third, we did not adjust for vitamin D status. This may be relevant because low vitamin D has been associated with frailty and with SLE susceptibility or disease activity in prior studies. However, vitamin D was not included in our prespecified main model, and a single baseline measurement may be influenced by season, supplementation, and other time-varying factors. Therefore, residual confounding related to vitamin D cannot be excluded. Fourth, while high-throughput proteomic platforms offer broad coverage, they remain constrained by panel design and assay sensitivity. Fifth, although MR was used to provide orthogonal genetic support for the proteomic findings, causal interpretation should remain cautious because some proteins were instrumented by only a small number of cis-pQTLs, which limited the scope and informativeness of standard MR sensitivity analyses. Sixth, we also note that routine SLE-related immunological markers, such as anti-dsDNA titre, C3 and C4, were not available in the UK Biobank dataset used in this study. As a result, we were unable to evaluate serological activity or immunological phenotypes of SLE, and our analyses focused on incident SLE risk rather than clinical disease activity or laboratory-defined disease severity. Seventh, we acknowledge that the mean age of incident SLE in our cohort (58 years) was older than expected for the general SLE population. This is likely related to the UK Biobank design, which enrolled participants aged 40–69 years at baseline. Therefore, our incident cases mainly reflect middle-aged and older-onset SLE, and the observed frailty associations are most directly generalisable to this age range rather than to younger patients with SLE. The relatively higher proportion of male SLE cases may likewise reflect the older age structure of the cohort, as men with SLE tend to have a later age at onset than women. Finally, residual confounding – particularly from unmeasured environmental or immunological variables – cannot be fully excluded, despite rigorous sensitivity analyses.

## Conclusion

In summary, our findings indicate that frailty is associated with a higher long-term risk of incident SLE, with stronger associations observed among individuals with greater genetic susceptibility. Circulating inflammatory markers appear to partially mediate this relationship, although the magnitude of mediation is modest. Future studies should explore whether modifying frailty can serve as a preventive or therapeutic strategy for SLE. If effective, incorporating frailty screening into routine care may facilitate earlier identification and more personalised management of high-risk individuals – particularly those with elevated genetic risk, who may be closer to the threshold at which frailty contributes to SLE onset than those with lower genetic susceptibility.

## CRediT authorship contribution statement

**Peizhi Deng:** Writing – review & editing, Writing – original draft, Visualization, Validation, Supervision, Software, Resources, Project administration, Methodology, Investigation, Formal analysis, Data curation, Conceptualization. **Siyu Yan:** Formal analysis, Data curation, Conceptualization. **Jianyun Lu:** Writing – review & editing, Funding acquisition, Formal analysis, Data curation, Conceptualization.

## Ethics approval and consent to participate

This study was conducted using the UK Biobank Resource under application number 224336 and was ethically approved by the NHS North West Multi-centre Research Ethics Committee (reference: 11/NW/0382). All participants provided written informed consent.

## Funding

This work was supported by grants from the 10.13039/501100001809National Natural Science Foundation of China (nos. 82504283 and 82574011) and Tibet Autonomous Region Science and Technology Project (no. XZ202402ZY0002). The funder had no role in studying design, data collection, analysis, reporting or the decision to submit for publication.

## Declaration of competing interest

The authors declare that they have no known competing financial interests or personal relationships that could have appeared to influence the work reported in this paper.

## Data Availability

The data were available from the UK Biobank Resource (www.ukbiobank.ac.uk/) and are available with permission via application number 224336. All other data supporting the findings in this study are available within the published articles or additional files.
