# Supplementary material for: Frailty phenotype, genetic risk and long-term incident systemic lupus erythematosus risk: Insights from the UK Biobank study integrated with multi-omics analysis
Source: Clin Med (Lond). 2026 Jun 4;26(4):100598. doi: 10.1016/j.clinme.2026.100598 (PMC13314888; doi:10.1016/j.clinme.2026.100598)
Supplement: Supplementary file 1 — Supplementary material [file mmc1.pdf]

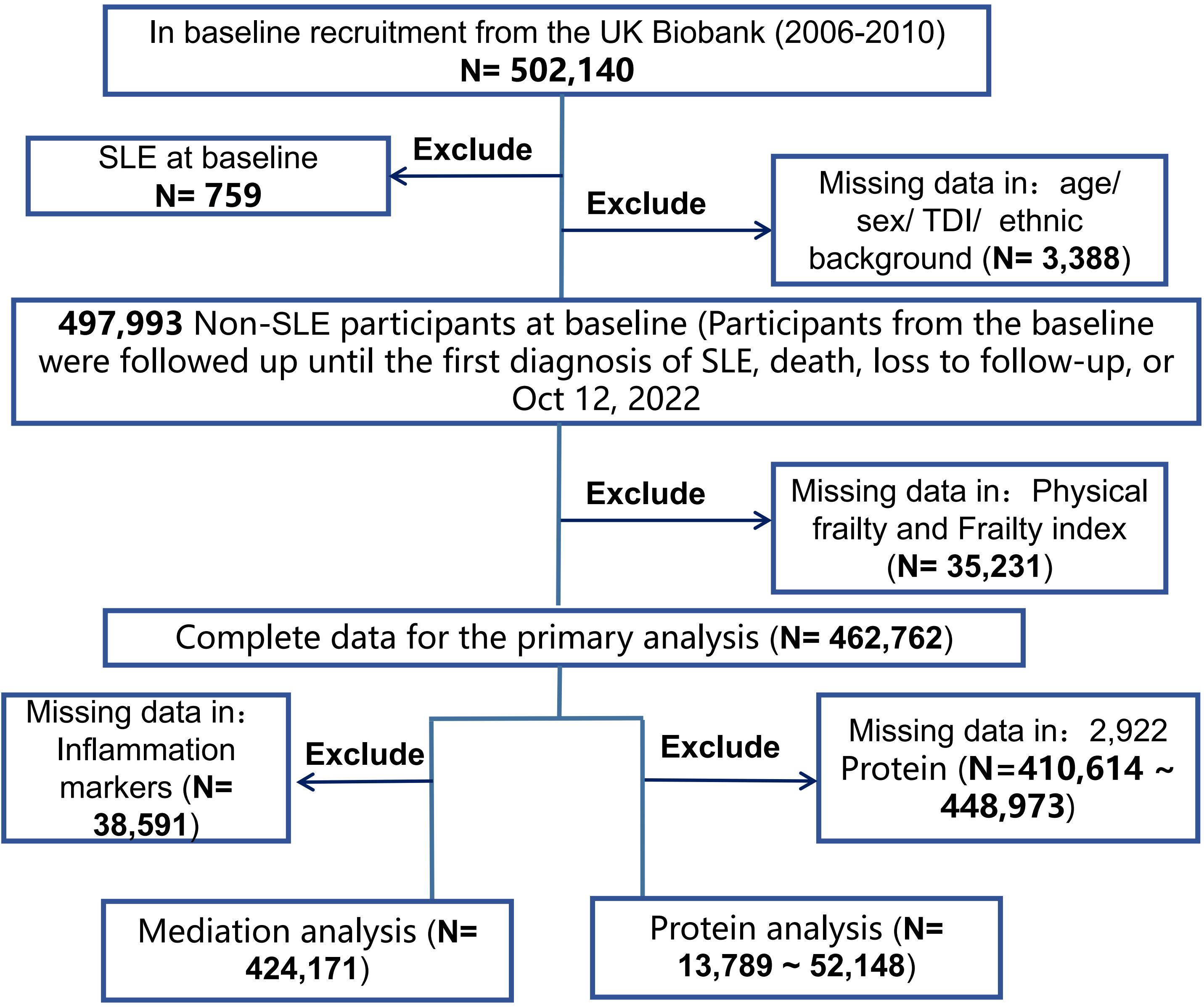

Figure S1. Flowchart of participants included in the UK biobank populations.

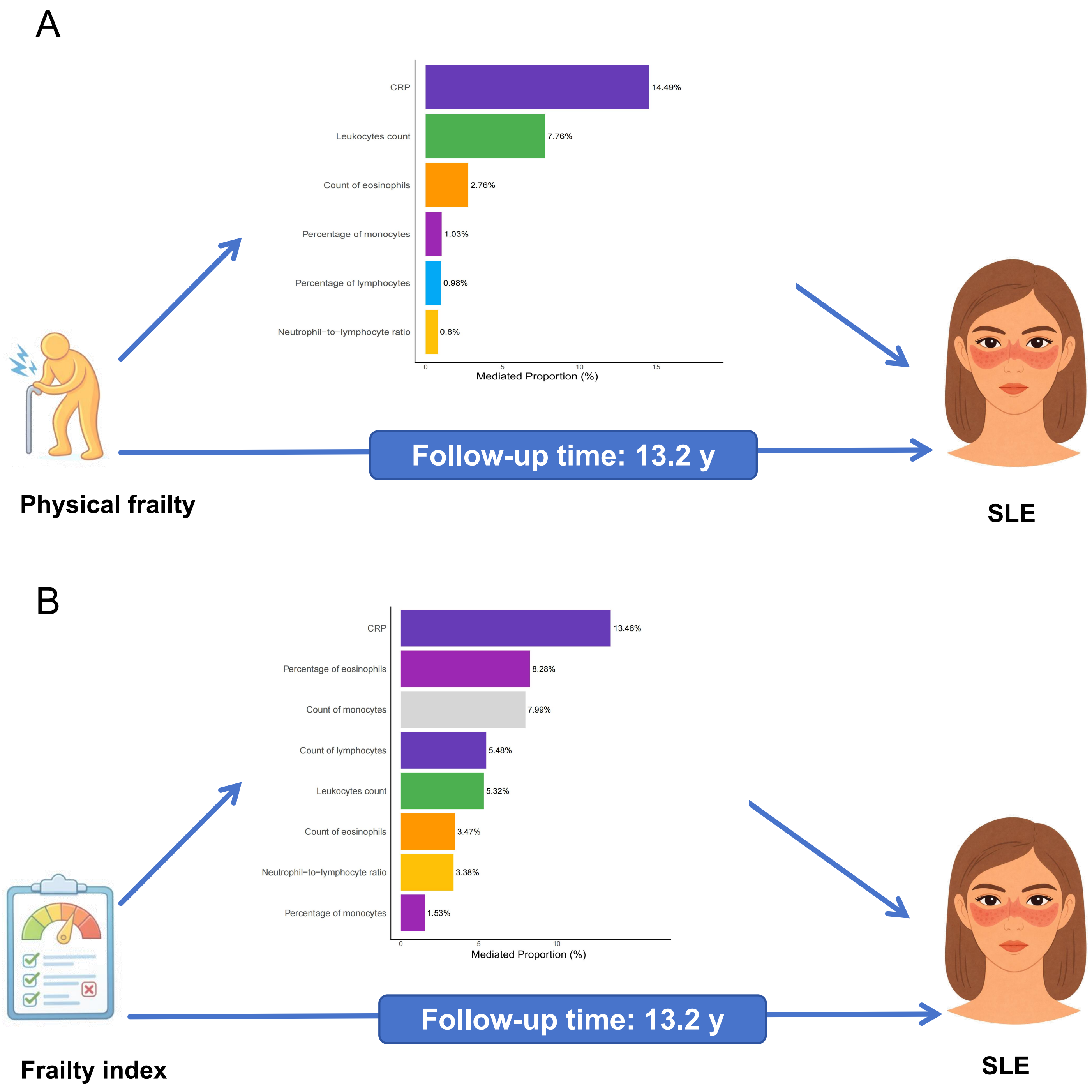

Figure S2. Inflammatory markers significantly mediated the prospective association between frailty and SLE incidence while adjusting for model 1 and multiple comparisons.
